# Positive Selection in East Asians for an *EDAR* Allele that Enhances NF-κB Activation

**DOI:** 10.1371/journal.pone.0002209

**Published:** 2008-05-21

**Authors:** Jarosław Bryk, Emilie Hardouin, Irina Pugach, David Hughes, Rainer Strotmann, Mark Stoneking, Sean Myles

**Affiliations:** 1 Department of Evolutionary Genetics, Max Planck Institute for Evolutionary Anthropology, Leipzig, Germany; 2 Institute of Biochemistry, Molecular Biochemistry, Medical Faculty, University of Leipzig, Leipzig, Germany; University of California, Berkeley, United States of America

## Abstract

Genome-wide scans for positive selection in humans provide a promising approach to establish links between genetic variants and adaptive phenotypes. From this approach, lists of hundreds of candidate genomic regions for positive selection have been assembled. These candidate regions are expected to contain variants that contribute to adaptive phenotypes, but few of these regions have been associated with phenotypic effects. Here we present evidence that a derived nonsynonymous substitution (370A) in *EDAR*, a gene involved in ectodermal development, was driven to high frequency in East Asia by positive selection prior to 10,000 years ago. With an in vitro transfection assay, we demonstrate that 370A enhances NF-κB activity. Our results suggest that 370A is a positively selected functional genetic variant that underlies an adaptive human phenotype.

## Introduction

Humans expanded within and out of Africa between 50,000 and 100,000 years ago and now inhabit radically different physical and cultural environments around the globe. A long-standing question in anthropology has been: How have humans genetically adapted to these environments? Numerous recent studies have attempted to address this issue for a review, see [1]. Most of these studies aim to identify genomic regions that have experienced local positive selection (i.e. geographically-restricted positive selection) from genome-wide polymorphism data e.g. [2], [3], [4]. A small number of these genomic regions are associated with phenotypes that have long been candidates for local positive selection, such as lactose tolerance [5], [6] and skin pigmentation [7]–[12]. For the large majority of these regions, however, no association to a locally adaptive phenotype has been established and the precise location of functional adaptive variants remains elusive. Moreover, sexual selection may be a plausible explanation for at least some of these candidate regions. Further investigation of candidate regions, including the possible functional consequences of the genetic variation observed in such regions, is required to fully understand the impact of local positive selection on our species. Here, we investigate one candidate gene, *EDAR*, in detail.

A strong signature of positive selection in East Asians has been found in the genomic region containing the *EDAR* gene (HGNC∶2895) according to tests based on the allele frequency spectrum [4], [13], [14], haplotype structure [2], [3] and population differentiation [15]–[19]. *EDAR* is known to be involved in the development of hair follicles, teeth and sweat glands [20] and harbors a nonsynonymous single nucleotide polymorphism (SNP) (rs3827760) that results in a valine to alanine substitution at position 370 of the amino acid sequence (V370A). Here we test the function of V370A in vitro and provide several lines of evidence suggesting that there was positive selection on the derived 370A allele in East Asians prior to 10,000 years ago.

## Results

### Fst analysis

**Figure 1:**
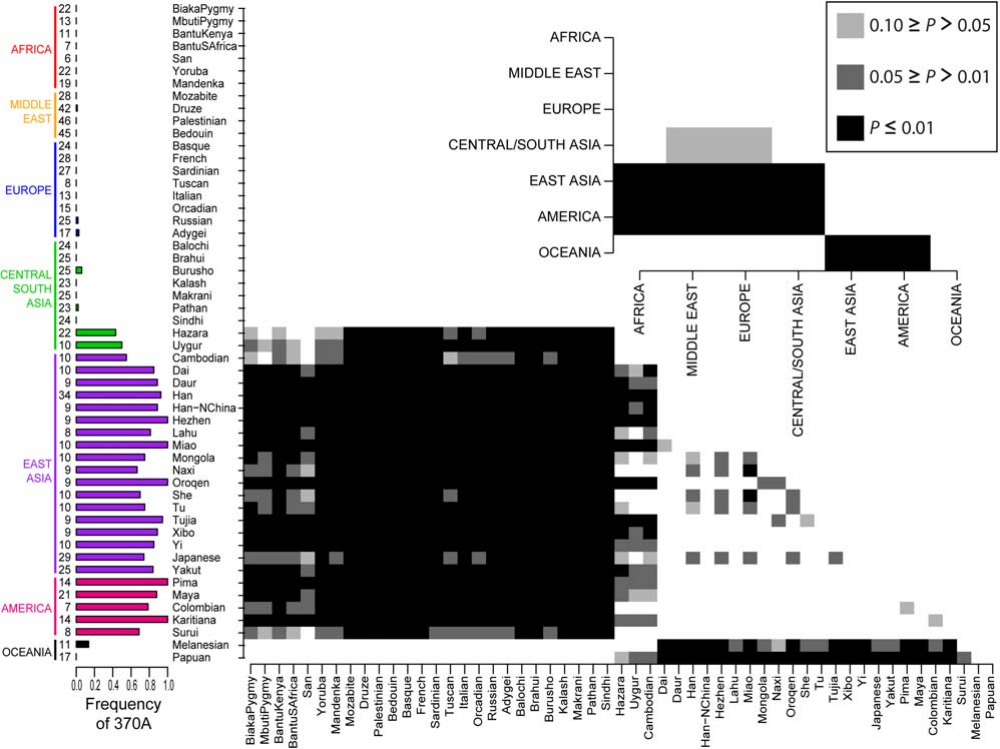
Worldwide allele frequencies and population differentiation for V370A. The vertical bar chart displays the frequency of the 370A allele in each of the populations represented in the CEPH-HGDP panel with sample sizes (number of individuals) on the left. The shaded boxes in the 53×53 and 7×7 matrices show which pairwise Fst values are significant compared to the empirical distribution at three *P* value thresholds (see the boxed-in *P* value legend).

We assessed worldwide population differentiation for V370A by use of the Fst statistic [21]. Alleles that have been targets of local positive selection tend to have unusually high Fst values [22]–[24]. We genotyped the V370A polymorphism in the 53 worldwide populations of the CEPH Human Genome Diversity Panel CEPH-HGDP; [25] and compared Fst values for V370A to an empirical Fst distribution derived from 2750 autosomal markers (2540 SNPs [26] and 210 indels [27]) previously typed in the same set of samples. Global Fst, the degree of differentiation among all 53 populations, for V370A is 0.760. This value is higher than all Fst values from the empirical global Fst distribution. To examine the patterns of population differentiation at a more refined geographical scale, we calculated Fst for every pairwise comparison among the 53 populations and 7 geographic regions to produce 53×53 and 7×7 Fst matrices, respectively. Each pairwise Fst value for V370A was then compared to the corresponding empirical distribution of pairwise Fst values to generate a *P* value. Worldwide allele frequencies and the *P* value matrices from the Fst analysis are depicted in Figure 1.

### Functional assay

**Figure 2:**
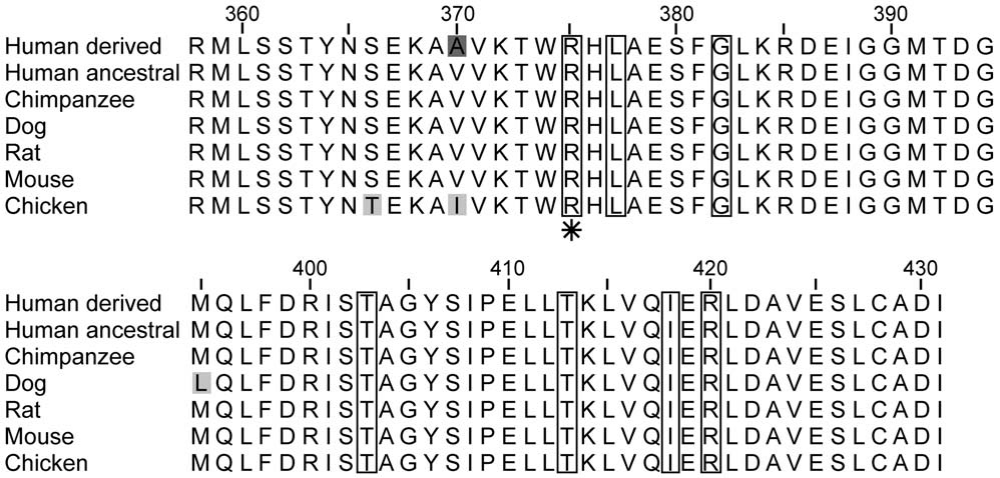
Multiple species alignment of the death domain of EDAR. The derived 370A allele is shaded in dark grey. Variable positions in the alignment are shaded in light grey. Sites at which nonsynonymous substitutions are known to result in hypohidrotic ectodermal dysplasia are boxed in. An asterisk indicates the site at which an Arg-His substitution causes decreased activation of NF-κB [28].

**Figure 3:**
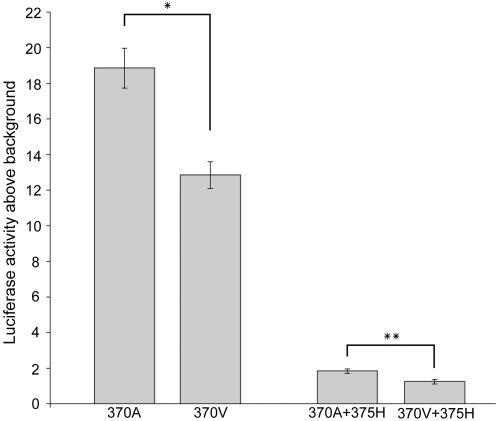
The derived 370A allele of EDAR results in enhanced activation of NF-κB in vitro. Transfection of a HEK293 cell line with ancestral (370V) and derived (370A) versions of EDAR activate NF-κB to a different extent on both a normal genetic background (comparison on the left) and on a background containing a known disease mutation (375H; comparison on the right). Luciferase activity is driven by EDAR-activated NF-κB. Data was averaged from two independent experiments with reads from at least 9 wells total for each clone. Error bars are standard error of the mean (* p<0.05, ** p<0.01).

EDAR (Swiss-Prot∶Q9UNE0) is a cell-surface receptor that, upon binding to its ligand, induces an intracellular cascade leading to the activation of NF-κB, a transcription factor [20]. To investigate the functional consequences of the V370A polymorphism, we measured NF-κB activation in vitro using a luciferase reporter assay, from a HEK293 cell line heterologously expressing the 370V and 370A variants of *EDAR* cDNA. As a positive control, we also performed the same transfections with 370V and 370A *EDAR* cDNAs that harbored an additional disease mutation (375H; Figure 2) that had previously been shown to severely reduce NF-κB activation in vitro [28]. In agreement with previous work, the cDNAs carrying 375H showed a ∼6 fold reduction in activation of NF-κB compared to cDNAs without 375H (Figure 3). Moreover, the derived 370A allele results in increased activation of NF-κB compared to the ancestral 370V allele on both the normal (p = 0.018, univariate ANOVA with experiment as a random factor and clone as a fixed factor) and the disease background (p<0.004, two-tailed t-test assuming equal variances; Figure 3).

### Estimation of time since fixation

**Figure 4:**
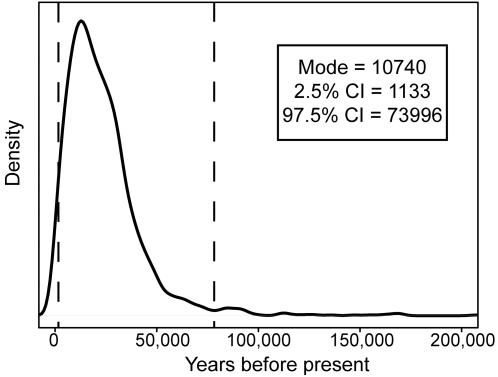
Density plot of the posterior distribution of estimates of the time since fixation of the 370A allele. The estimated time since fixation of the 370A allele is the mode of the distribution (10740 years before present). The 2.5% and 97.5% confidence intervals are boxed in and are indicated by the hashed vertical lines.

Finally, we assessed the timing of the selection event on 370A from ∼22 kb of *EDAR* sequence from 23 individuals of Chinese ancestry from the Seattle SNPs data set (http://pga.gs.washington.edu/). We employed a method that estimates the time since fixation of a beneficial allele [29]. Only one chromosome from the data set carried the ancestral 370V allele and we removed it from the analysis and assumed that the 370A allele had reached fixation. The resulting estimate for the time since fixation is 10,740 years (95% CI = 1133–73996; Figure 4).

## Discussion

Recent scans of genome-wide polymorphism data have generated long lists of genomic regions that are believed to have been targeted by local positive selection, but few of these regions have been shown to harbor functional variants or to be linked to a putatively adaptive phenotype. Detailed investigation of these candidate genomic regions is required for a comprehensive picture of local human adaptation. The present study provides an analysis of genetic variation and function at the *EDAR* gene, a candidate for positive selection in East Asians identified from recent genome scans [2]–[4], [13]–[16], [19].

We provide two lines of evidence supporting the hypothesis that the derived 370A allele in *EDAR* is functional and experienced positive selection in East Asians. First, worldwide population differentiation for 370A as measured by Fst is highly unusual. Figure 1 demonstrates that 370A has a highly unusual worldwide frequency distribution, supporting a scenario in which the 370A allele was driven to high frequency in East Asians and Native Americans by positive selection (Figure 1). Two Central/South Asia populations, the Uygur and the Hazara, have intermediate frequencies of 370A (0.44 and 0.5, respectively), in agreement with their close genetic relationship to East Asians [30], [31]. The 370A allele is also found at low frequency in Melanesia (0.12), and was likely introduced there via the recent Austronesian expansion [32]. Otherwise 370A is absent in Africans and Papuans and is observed at very low frequency in most of Central/South Asia, Europe and the Middle East (Figure 1).

The second line of evidence suggesting an adaptive functional role for 370A stems from its location in the amino acid sequence of EDAR. V370A is located in the death domain, a protein interaction module, of EDAR. The death domain of EDAR is highly conserved (Figure 2) and interacts with the death domain of EDARADD, an intracellular ligand to EDAR [33], [34]. This interaction initiates an intracellular signalling cascade that results in the activation of the transcription factor NF-κB [35]. Therefore, V370A may alter binding affinity with the death domain of EDARADD and thereby influence the activation of NF-κB. Moreover, seven nonsynonymous substitutions in the death domain of EDAR cause hypohidrotic ectodermal dysplasia (HED) in humans [36], a disease characterized by sparse and thin hair, missing teeth and the absence of sweat glands (OMIM∶604095; see Figure 2). In particular, the R375H substitution, only 5 amino acids upstream from V370A, results in a loss of affinity for EDARADD and reduced NF-κB activation [28]. We therefore tested the function of V370A in vitro and found that the 370A allele does differ from the ancestral 370V allele in that it results in enhanced NF-κB activation (Figure 3).

Our results contradict a recent report from Fujimoto et al. [37] in which the 370A allele was shown to reduce NF-κB activation in vitro [37]. We are confident that our results are correct for two reasons. First, in addition to observing enhanced NF-κB activation for the derived 370A allele on a normal genetic background, we measured NF-κB activation of the 370V and 370A alleles on the background of a disease mutation (375H) that was previously demonstrated to result in significantly reduced NF-κB activation [28]. In agreement with [28], we observed significantly reduced NF-κB activation in both clones carrying the 375H disease mutation. Moreover, NF-κB activation was significantly higher in the derived 370A+375H construct than in the ancestral 370V+375H construct (Figure 3). Thus, we observed enhanced NF-κB activation for the derived 370A allele in two independent constructs. Second, East Asians have thicker hair than Europeans and Africans [38] and an increase in NF-κB activation is arguably more likely to lead to the thicker East Asian hair phenotype. This is because a decrease in NF-κB activation, as observed for carriers of the 375H allele that causes hypohidrotic ectodermal dysplasia, is associated with thin hair. We suspect that the large doses of plasmid DNA (300 ng) and long post-transfection incubation period (48 h) could have induced cell death in the experiments of Fujimoto et al. [37]. Although EDAR-induced cell death is a matter of controversy [39], [40], several features of cell death (detachment, rounding and membrane permeation) are observed 36 hours after transfection with a high dosage of plasmid (500 ng) [39]. Control cells transfected with the same amount of an empty vector do not display features of cell death, suggesting that simply overdosing the cells with plasmid is not responsible for cell death [39]. Thus, we speculate that the reduced NF-κB activation from 370A observed by Fujimoto et al. [37] could be the result of induced cell death and that 370A in fact enhances NF-κB activation.

Our estimate of the time since fixation of 370A in a sample of 45 Chinese chromosomes is 10,740 years (Figure 4). This estimate involves several assumptions (see Materials and Methods) and should be interpreted with caution. Nevertheless the result suggests that 370A was likely at high frequency before the colonization of the Americas 10,500–14,000 years ago [41]–[43]. Thus, the high frequency of 370A in Native Americans (see Figure 1) is most likely due to positive selection prior to migrations from Asia to America.

In summary, we have demonstrated that the worldwide frequency distribution of 370A is highly unusual and that 370A was likely rising in frequency by positive selection in East Asia prior to 10,000 years ago. In addition, we have shown that the 370A allele results in enhanced NF-κB activation in vitro. What was the source of the selection pressure on 370A and what effect may 370A have on the phenotype? Since EDAR is involved in ectodermal development, 370A might be expected to affect teeth, hair, skin, nails and/or sweat glands. Fujimoto et al. [37] recently noted an association between 370A and hair thickness. Replication of this result is desirable since correction for population structure was inadequate: only a single SNP was used to correct for population structure. Nevertheless, the results of Fujimoto et al. [37] are suggestive, especially since East Asians have thicker hair than Europeans and Africans [38]. These observations lead us to question why thicker hair may have been advantageous in ancestral East Asian environments. Of course, thicker hair may not have been adaptive at all and may simply be the result of phenotypic hitchhiking: selection on 370A may have targeted a different phenotype (e.g. tooth morphology [16]) and hair thickness may have resulted as a by-product of this selection. Sexual selection also remains a possibility.

Regardless of the nature of the selective force, our results provide compelling evidence that positive selection has acted on the 370A allele in *EDAR*. In addition, our finding that 370A results in increased NF-κB activation suggests further lines for investigation: in particular, how does this increased NF-κB activation influence the expression of the target genes regulated by NF-κB? Such future studies will lead to a more complete understanding of the phenotypic effect of 370A, and will permit more explicit tests of hypotheses concerning the possible selection pressure(s) responsible for its rapid increase in frequency in East Asia.

## Methods

### Experimental Procedures

We genotyped the V370A polymorphism (rs3827760) in the HGDP-CEPH Human Genome Diversity Cell Line Panel [25] using the SNaPshot™ minisequencing kit (Applied Biosystems, Foster City, CA). Atypical and related individuals were removed [44], which resulted in 952 individuals from 53 populations with 10 missing genotypes. One population (Burusho) was out of Hardy-Weinberg equilibrium (p = 0.008), but this value becomes non-significant after correction for multiple comparisons. We obtained an empirical Fst distribution from 2750 autosomal markers (2540 SNPs [31] and 210 indels [32]) previously typed in 927 individuals from the CEPH-HGDP panel. Fst values for V370A were calculated from the same set of 927 individuals to allow for an unbiased comparison to the empirical distribution. Each *P* value was calculated as the proportion of Fst values from the empirical distribution that were greater than or equal to the observed Fst value.

Conservation of *EDAR* [NP_071731.1] was assessed by aligning all orthologs of *EDAR* obtained from release 52 of Homologene (http://www.ncbi.nlm.nih.gov/entrez/query.fcgidbhomologene, Homologene ID = 7699) using MUSCLE version 3.6 [45].

We analysed 22094 bp of resequencing data from the *EDAR* gene from 23 individuals of Asian origin (http://pga.mbt.washington.edu/) to investigate the age of the selective event. The method [29] assumes that the selective sweep has reached fixation, so the single non-derived haplotype was removed, resulting in 45 chromosomes for analysis. The method also assumes that the sequence data is contiguous, but the available data included 7 sequenced regions distributed across 98136 bp of the genome. We therefore concatenated the 7 regions to produce one contiguous sequence of 22094 bp. Simulations were performed to obtain a sample from the posterior distribution of the time since fixation (T), conditional on the number of segregating sites (17), Tajima's D (−2.13) and the number of haplotypes (13). The following parameters were used: μ = 2.3×10^−8^ [46], [47]; ρ = 0.001 [46], [48]; N_e_ = 10,000 [49], and a generation time of 20 years. As the observed number of haplotypes is affected by recombination events across the ∼98 kb of *EDAR* sequence, we increased ρ by a factor of 5 in our simulations of ∼22 kb of sequence. We found, however, that point estimates and confidence intervals did not vary widely when different values of ρ, ranging from 0.0001 to 0.005, were used (data not shown). We used the mode of the distribution of T as the point estimate of the time since fixation as recommended in [29].

*EDAR* cDNA was generated from human fetal skin total RNA (Stratagene) using oligo-dT primers and SuperScript II reverse transcriptase (Invitrogen) and amplified with primers covering amino acid positions 31–448. The PCR product was cloned into the pCMV-Tag2c vector (Stratagene) and several inserts were sequenced to determine the allelic variant of *EDAR* corresponding to amino acid position 370. Clones resulting in valine or alanine at this position were termed p370V and p370A, respectively. The clones underwent site-directed mutagenesis using the QuikChange II Kit (Stratagene) to generate the disease variant with histidine at position 375 on both ancestral and derived backgrounds (p370V+375H and p370A+375H). Whole inserts in mutated clones were sequenced to ensure correctness.

For the in vitro functional study, HEK293 cells (RZPD, Germany) were seeded in a 96-well plate at a density of 5×10^4^ cells/well and cultured in DMEM supplemented with 10% (vol) FBS (Invitrogen). After approximately 48 hours, cells were transfected with 50 ng/well of total DNA, consisting of equal amounts (ng) of 370V or 370A (each with or without disease variant), pNF-κB-Luc vector (Clonetech) and pRL4.74 vector (Promega). Transfection was performed using FuGene HD reagent (Roche) diluted with OptiMEM medium (Invitrogen) in 100 µl total volume in 5 replicates for each version of the *EDAR* construct. Cells transfected with empty pCMV-Tag2 vector were used as a background signal control. After 18 h, luciferase signals were read using the Dual-Glo Luciferase Assay System (Promega) and a Victor-2 luminometer (PerkinElmer). The signal from pNF-κB-Luc was normalized to the signal from pRL4.74 and the background was subtracted. An 18 h timepoint was selected based on previous time-curve experiments (data not shown).
